# Comparing different scoring systems for predicting mortality risk in preterm infants: a systematic review and network meta-analysis

**DOI:** 10.3389/fped.2023.1287774

**Published:** 2023-12-15

**Authors:** Zhaolan Zeng, Zeyao Shi, Xiaowen Li

**Affiliations:** ^1^ Department of Neonatology Nursing, West China Second University Hospital, Sichuan University/West China School of Nursing, Sichuan University, Chengdu, China; ^2^ Key Laboratory of Birth Defects and Related Diseases of Women and Children (Sichuan University), Ministry of Education, Chengdu, China

**Keywords:** critical illness scoring system, preterm infants, mortality risk, predictive efficiency, network meta-analysis

## Abstract

**Background:**

This study aimed to compare the predictive values of eight scoring systems (Neonatal Critical Illness Score [NCIS], Neonatal Therapeutical Intervention Score System [NTISS], Clinical Risk Index for Babies [CRIB], Clinical Risk Index for Babies II [CRIB-II], Score for Neonatal Acute Physiology Perinatal Extension [SNAPPE], Score for Neonatal Acute Physiology Perinatal Extension II [SNAPPE-II], Score for Neonatal Acute Physiology [SNAP], and Score for Neonatal Acute Physiology II [SNAP-II]) for the mortality risk among preterm infants.

**Methods:**

The Embase, PubMed, Chinese Biomedical Database, Web of Science, and Cochrane Library databases were searched to collect studies that compared different scoring systems in predicting the mortality risk in preterm infants from database inception to March 2023. Literature screening, data extraction, and bias risk assessment were independently conducted by two researchers. Subsequently, the random-effects model was used for the network meta-analysis.

**Results:**

A total of 19 articles were included, comprising 14,377 preterm infants and 8 scoring systems. Compared to CRIB-II, NCIS, NTISS, SNAP-II, and SNAPPE-II, CRIB demonstrated better predictive efficiency for preterm infant mortality risk (*P < *0.05). Relative to CRIB, CRIB-II, and SNAPPE, SNAP-II had worse predictive efficiency for preterm infant mortality risk (*P *< 0.05). The surface under the cumulative ranking curve of the eight scoring systems was as follows: CRIB (0.980) > SNAPPE (0.718) >SNAP (0.534) >CRIB-II (0.525) >NTISS (0.478) >NCIS (0.422) >SNAPPE-II (0.298) >SNAP-II (0.046).

**Conclusion:**

The CRIB scoring system showed the highest accuracy in predicting preterm infant mortality risk and was simple to perform. Therefore, CRIB selection can be prioritized in clinical practice.

**Systematic Review Registration:**

https://www.crd.york.ac.uk/prospero/display_record.php?RecordID=434731, PROSPERO (CRD42023434731).

## 1. Introduction

According to the World Health Organization, approximately 15 million preterm infants (gestational age <37 weeks) are born every year worldwide. The death rate of preterm infants accounts for approximately 35% of neonates and 16% of children aged <5 years. Premature birth is an important global health problem (1, 2). Immature organ function and poor adaptability to the extrauterine environment in preterm infants make them prone to asphyxia, acute respiratory distress syndrome, intracranial hemorrhage, infection, and other complications that increase the risk of death. Approximately 1.27 million babies died of complications related to preterm delivery worldwide in 1990. With the progress in perinatal medicine and neonatal intensive care technology, the survival rate of preterm babies has gradually increased. However, the data showed that 0.66 million babies still died of preterm birth-related complications worldwide in 2019 (3).

Studies indicate that timely and effective disease assessment and mortality risk prediction may greatly reduce clinical mortality among preterm infants (4). The Neonatal Critical Illness Score (NCIS), Neonatal Therapeutical Intervention Score System (NTISS), Clinical Risk Index for Babies (CRIB), Clinical Risk Index for Babies II (CRIB-II), Score for Neonatal Acute Physiology Perinatal Extension (SNAPPE), Score for Neonatal Acute Physiology Perinatal Extension II (SNAPPE-II), Score for Neonatal Acute Physiology (SNAP), and Score for Neonatal Acute Physiology II (SNAP-II) are commonly used in clinics and are reliable in predicting the mortality risk of preterm infants. To date, no reviews have explored the strengths and weaknesses of the different scoring systems (5). Therefore, this study aimed to compare the predictive efficiency of the above critical illness scoring systems through a network meta-analysis and estimate the rank order of each scoring system. This study provides reference for clinical decision-making and as such may further reduce the clinical mortality of preterm infants.

## 2. Methods

The study protocol for this systematic review and meta-analysis was prospectively registered with PROSPERO (CRD42023434731).

### 2.1. Search strategyy

Two independent researchers (ZLZ and ZYS) systematically searched for original research articles in Embase, PubMed, Web of Science, Cochrane Library, and Chinese Biomedical Database, from the inception of each database to March 2023, with the languages restricted to Chinese and English. The following search terms were used: “[(premature OR preterm infant OR low birth weight OR LBW OR very low birth weight OR VLBW) AND (neonatal critical illness score OR NCIS OR neonatal therapeutical intervention score system OR NTISS OR clinical risk index for babies OR CRIB OR clinical risk index for babies Ⅱ OR CRIB-Ⅱ OR score for neonatal acute physiology perinatal extension OR SNAPPE OR score for neonatal acute physiology perinatal extension Ⅱ OR SNAPPE-Ⅱ OR score for neonatal acute physiology OR SNAP OR score for neonatal acute physiology Ⅱ OR SNAP-Ⅱ)].” The Web of Science search strategy is presented in detail in Supplementary Table S1. Similar search threads were used for all other databases. Reference lists of relevant literature were reviewed to identify as many available studies as possible.

### 2.2. Selection criteria

The inclusion criteria were as follows: (1) retrospective or prospective cohort study, (2) research participants were preterm infants (gestational age <37 weeks), and (3) the predictive value of any two or more scoring systems among the NCIS, SNAP, SNAP-II, SNAPPE, SNAPPE-II, CRIB, CRIB-II, and NTISS on the mortality risk in preterm infants were compared. The exclusion criteria were: (1) repeatedly published literature; (2) literature reviews, case reports, conference abstracts, and gray literature; and (3) studies with incomplete data.

### 2.3. Screening process and data extraction

Two researchers (ZLZ and ZYS) independently screened the articles, extracted the data, and cross-checked the results. Any discrepancies were resolved through discussions with a third researcher (XWL). First, the titles and abstracts were reviewed for relevance, and then two researchers independently read the full texts of all potential articles to determine the final studies for inclusion. The following data were extracted: title, author, country, sample size, gestational age, birth weight, study type, scoring system, and outcome indicator.

### 2.4. Risk of bias assessment

Two researchers (ZLZ and ZYS) independently assessed the quality of the included studies and cross-checked the results. Differences were resolved by discussion or by asking a third researcher (XWL) to assist in the judgment. The risk of bias was assessed using the Newcastle-Ottawa Scale (NOS) based on three categories: selection, comparability, and outcome (6). Studies awarded six or more stars were classified as high quality.

### 2.5. Statistical analysis

Data were compiled and analyzed using Markov chain Monte Carlo simulation chains of R software (version 4.2.1) based on a Bayesian framework according to the PRISMA NMA instruction manual. Stata software (version 16.0) was used to draw the network and funnel plots, and verify the results of network meta analysis. The area under the curve (AUC) and their respective 95% confidence interval (CI) or standard error (SE) were used to combine the data. The *I^2^* test estimated heterogeneity between studies, where by *I^2 ^*< 50% was considered slight heterogeneity and *I^2 ^*≥ 50% was considered high heterogeneity. Due to potential differences between studies, we decided to use a random-effects model rather than a fixed-effects model to analyze the data, employing four Markov chains, 10,000 burn-in iterations, and 30,000 simulation iterations. To quantify and demonstrate the agreement between indirect and direct comparisons, we used the nodal method calculated according to the instructions in Stata. The consistency test was passed if the *P*-value was > 0.05. The potential scale-reduced factor (PSRF) was used to determine model convergence. When the PSRF value was <1.05, the convergence of the iterative effect was good. To rank the predictive value of each scoring system, we used the surface under the cumulative ranking (SUCRA) and mean ranks. The SUCRA values ranged from 0 to 1, and a larger SUCRA value implied a higher predictive value. Small-scale studies could lead to publication bias in NMA, for which we created network funnel plots and checked them visually using symmetry criteria.

## 3. Results

### 3.1. Study selection

**Figure 1:**
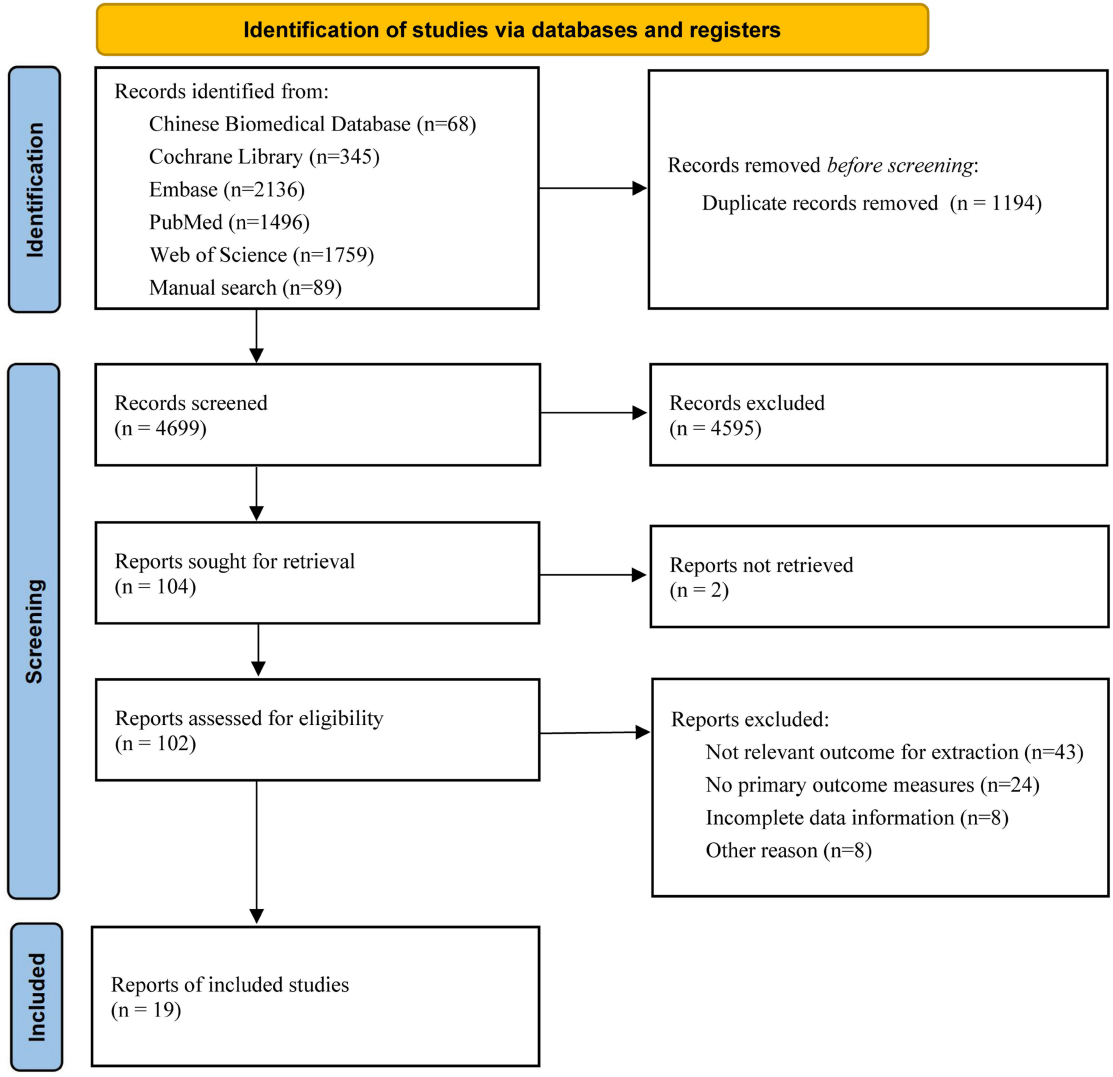
Flowchart of the study selection process.

A total of 5,804 studies were obtained in the initial search, of which 4,072 were found through the database search and 89 were obtained from other sources. Nineteen studies were finally included after screening according to the inclusion and exclusion criteria (7–25). The study selection process is illustrated in Figure 1.

### 3.2. Study characteristics

The 19 articles included in the study were published between 1994 and 2022, and comprised 14,377 preterm infants and 8 scoring systems: NCIS, SNAP, SNAP-II, SNAPPE, SNAPPE-II, CRIB, CRIB-II, and NTISS. Their main characteristics are shown in Table 1.

**Table 1:** Characteristics of the included studies.

Study	Year	Country	Sample (*n*)	Mortality	Gestational age (weeks)	Birth weight (g)	Participants	Study type	Scoring systems	NOS score
Yang et al. (7)	2022	China	192	40.63%	27.15 ± 2.07	838 ± 119	Admission age < 1 h, gestational age < 37 weeks	Retrospective	①③⑤⑥⑦	7
Vardhelli et al. (8)	2022	India	419	8.83%	29.84 ± 1.8	1,238 ± 376	Admitted to NICU within 24 h of birth and gestational age < 33 weeks	Prospective	⑤⑦	8
Sotodate et al. (9)	2022	Japan	171	11.11%	25.5 ± 1.6	741.3 ± 208.2	Infants with gestational age between 22 and 27 weeks	Retrospective	③⑤⑦	7
Yang et al. (10)	2022	China	223	16.59%	29.3 ± 1.8	1,179.9 ± 238.7	Admission age < 12 h of birth, gestational age < 37 weeks, birth weight <1500g	Retrospective	①③⑤⑥	7
Sokou et al. (11)	2021	Greece	224	/	32∼37	1,480∼2,580	Critically-ill neonates, gestational age < 37 weeks	Prospective	③④⑤	6
Hsu et al. (12)	2021	Taiwan	1,734	16.03%	27 ± 1.58	915 ± 194.1	Neonates on intubation for respiratory failure	Retrospective	⑤⑧	9
Dalili et al. (13)	2020	Iran	344	26.45%	23∼32	1,134 ± 325	Birth weight <1500 g or gestational age<32weeks	Prospective	⑤⑦	8
Rinta-Koski et al. (14)	2018	Finland	598	8.86%	28 ± 6	<1,500	Birth weight <1500g	Retrospective	③⑤	6
Guenther et al. (15)	2015	Germany	5,340	7.17%	28 ± 2	1,090 ± 232.5	Very low birth weight infants with a gestational age <33 weeks	Retrospective	⑥⑦	9
Reid et al. (16)	2015	Australia	1,607	8.53%	29 ± 2	1,250 ± 556.75	Gestational age < 32 weeks and admitted to NICU within 48 h of birth	Prospective	⑤⑦	8
Zhang (17)	2012	China	97	18.56%	30 ± 1.5	1,300 ± 200	Gestational age ≤ 32 weeks, birth weight <1500 g and admitted to NICU within 1 h of birth	Prospective	①⑤⑦	8
Phillips et al. (18)	2011	UK	408	11.52%	23∼37	370∼1,500	Birth weight <1501g	Retrospective	⑥⑦	6
Bührer et al. (19)	2008	Germany	1,358	8.98%	28 ± 4	1,060 ± 298.75	Very low birth weight infants (birth weight <1,500 g)	Retrospective	⑥⑦	7
De Felice et al. (20)	2005	Italy	147	11.56%	28.6 ± 2.5	1,070 ± 325	Gestational age ≤ 31 weeks and birth weight <1500g	Prospective	⑥⑦	8
Gagliardi et al. (21)	2004	Italy	720	16.67%	29 ± 2.25	1,090 ± 274.75	Birth weight <1500 g, gestational age 23–32 weeks	Prospective	⑤⑥⑦	8
Eriksson et al. (22)	2002	Sweden	218	17.89%	28 ± 1.8	1,083 ± 259	Birth weight <1500 g and gestational age ≤ 31 weeks	Retrospective	②④⑥⑧	9
Braga et al. (23)	1999	Portugal	169	21.30%	<37	540∼1,500	Birth weight <1500g	Retrospective	②④⑥⑧	6
Bastos et al. (24)	1997	Portugal	186	21.51%	30 ± 3.25	1,168 ± 277.5	Birth weight <1500 g and / or gestational age <32 weeks	Retrospective	②④⑥⑧	9
Rautonen et al. (25)	1994	Finland	222	22.97%	28 ± 3	1,015 ± 286.25	Birth weight <1500g	Retrospective	②④⑥	6

① NCIS; ② SNAP; ③ SNAP-II; ④ SNAPPE; ⑤ SNAPPE-II; ⑥ CRIB; ⑦ CRIB-II; ⑧ NTISS.

### 3.3. Risk of bias of included studies

Quality evaluation results showed that seven studies (8, 11, 13, 14, 18, 23, 25) did not describe the determination of exposure in detail; four studies (16, 17, 20, 21) did not describe the follow-up time; and nine studies (7, 9–11, 14, 18, 19, 23, 25) did not address the confounding factors. All included studies had a total quality score ≥6 points, suggesting that the quality of the included studies was high (Table 1).

### 3.4. Network plot

**Figure 2:**
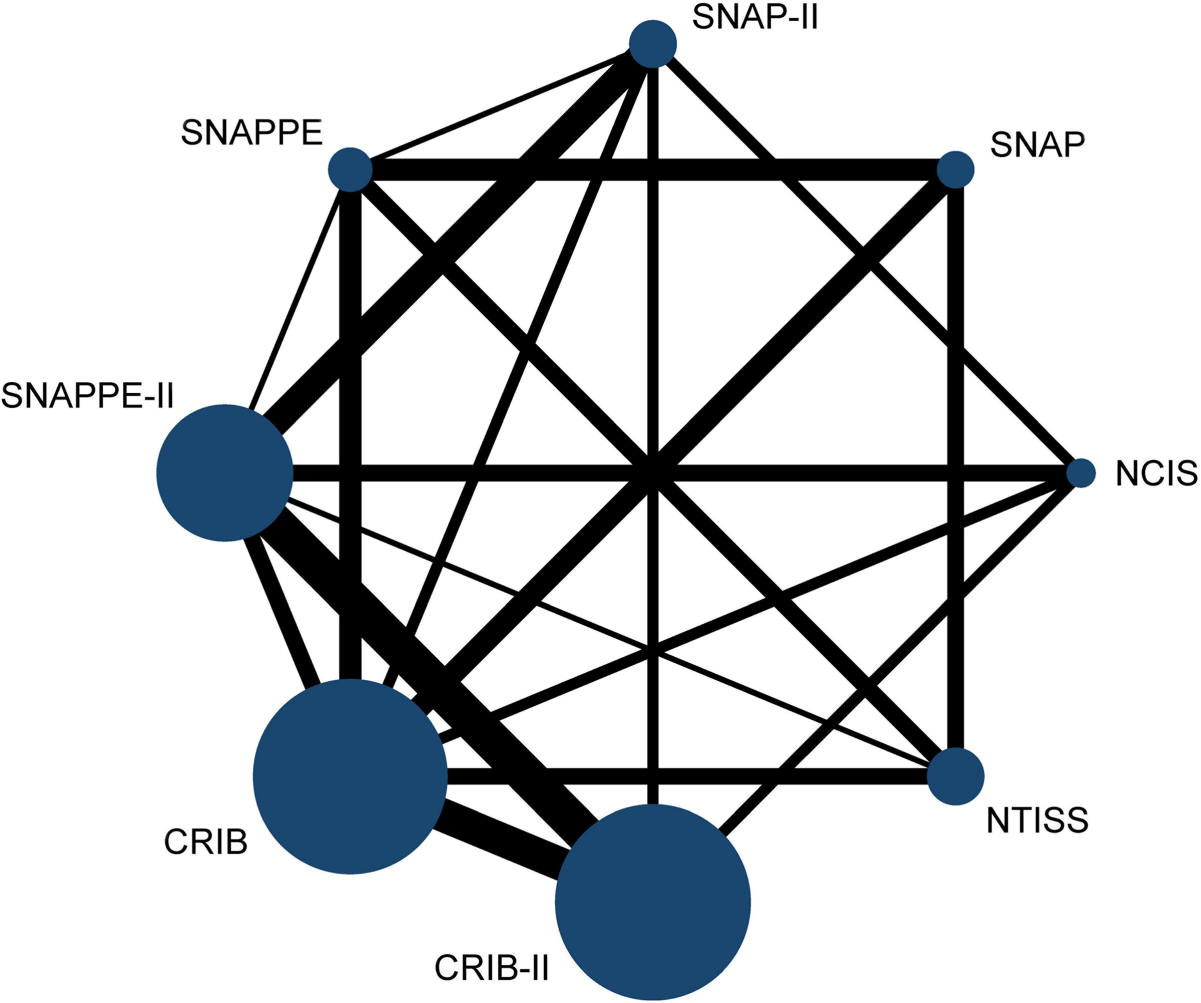
Network plot.

We described network diagrams for different scoring systems, In the presented network diagrams, each node represents a different scoring systems, and the lines connecting the nodes represent a direct comparison between scoring systems. The size of each node and the width of the connecting lines are proportional to the number of studies. The network plot is shown in Figure 2.

### 3.5. Model convergence

The Bayesian model constructed in this study showed that the fluctuation of the Markov chain was small, the iterative trajectory tended to be stable, and the PSRF value is close to 1, which indicates that the model converged better and could effectively predict the data. And the prediction results of R software and Stata software are consistent.

### 3.6. Assessment of heterogeneity and consistency

A heterogeneity test was performed on the original studies that directly compared each scoring system, and the results showed that the heterogeneity between CRIB and CRIB-II (*I^2 ^*= 58.3%) was high. There was slight heterogeneity between SNAPPE-II and SNAP-II (*I^2 ^*= 43.3%) and CRIB-II (*I^2 ^*= 18.2%); other studies had no heterogeneity. The node-split method was used to test the consistency. There was no significant difference between the indirect and direct comparison results of each scoring system (*P *> 0.05), indicating that the effects of consistency between the studies were acceptable.

### 3.7. Network meta-analysis results

The network meta-analysis showed that compared with CRIB-II, NCIS, NTISS, SNAP-Ⅱ, and SNAPPE-II, CRIB was more effective in predicting the mortality risk in preterm infants (*P *< 0.05). Relative to CRIB, CRIB-II, and SNAPPE, SNAP-Ⅱ was worse in predicting the mortality risk in preterm infants (*P *< 0.05). There were no statistically significant differences between the other scoring systems (*P *> 0.05), as shown in Table 2.

**Table 2:** Results of indirect comparisons of the scoring systems.

CRIB	–	–	–	–	–	–	–
0.053 (0.023, 0.087)	CRIB-Ⅱ	–	–	–	–	–	–
0.061 (0.004, 0.122)	0.008 (−0.049, 0.066)	NCIS	–	–	–	–	–
0.055 (0.009, 0.102)	0.002 (−0.052, 0.053)	−0.006 (−0.078, 0.064)	NTISS	–	–	–	–
0.050 (−0.001, 0.100)	−0.003 (−0.063, 0.053)	−0.011 (−0.088, 0.063)	−0.005 (−0.060, 0.049)	SNAP	–	–	–
0.101 (0.054, 0.159)	0.048 (0.002, 0.101)	0.040 (−0.022, 0.107)	0.045 (−0.013, 0.115)	0.051 (−0.013, 0.126)	SNAP-Ⅱ	–	–
0.034 (−0.014, 0.081)	−0.019 (−0.076, 0.034)	−0.027 (−0.103, 0.044)	−0.021 (−0.075, 0.031)	−0.016 (−0.070, 0.037)	−0.067 (−0.138, −0.005)	SNAPPE	–
0.070 (0.035, 0.108)	0.017 (−0.016, 0.048)	0.009 (−0.048, 0.064)	0.015 (−0.035, 0.066)	0.020 (−0.037, 0.079)	−0.030 (−0.080, 0.008)	0.036 (−0.018, 0.093)	SNAPPE-Ⅱ

### 3.8. Ranking of predictive value

**Figure 3:**
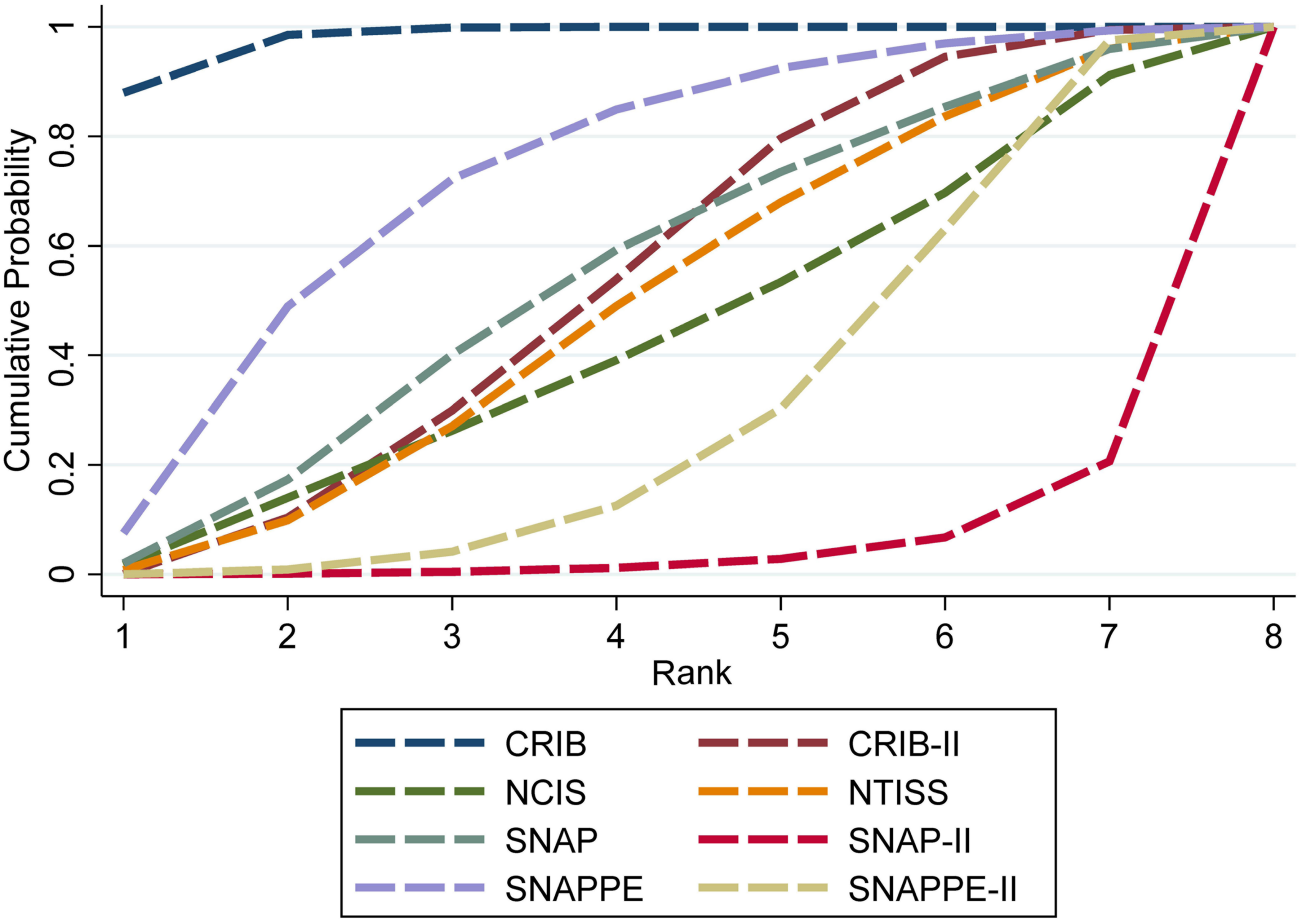
SUCRA curve of predictive value of the eight scoring systems.

The SUCRA values of the eight scoring systems for predicting mortality risk in preterm infants were as follows: CRIB (0.980) >SNAPPE (0.718) >SNAP (0.534) >CRIB-Ⅱ (0.525) >NTISS (0.478) >NCIS (0.422) >SNAPPE-Ⅱ (0.298) >SNAP-Ⅱ (0.046) (Figure 3).

### 3.9. Publication bias test

**Figure 4:**
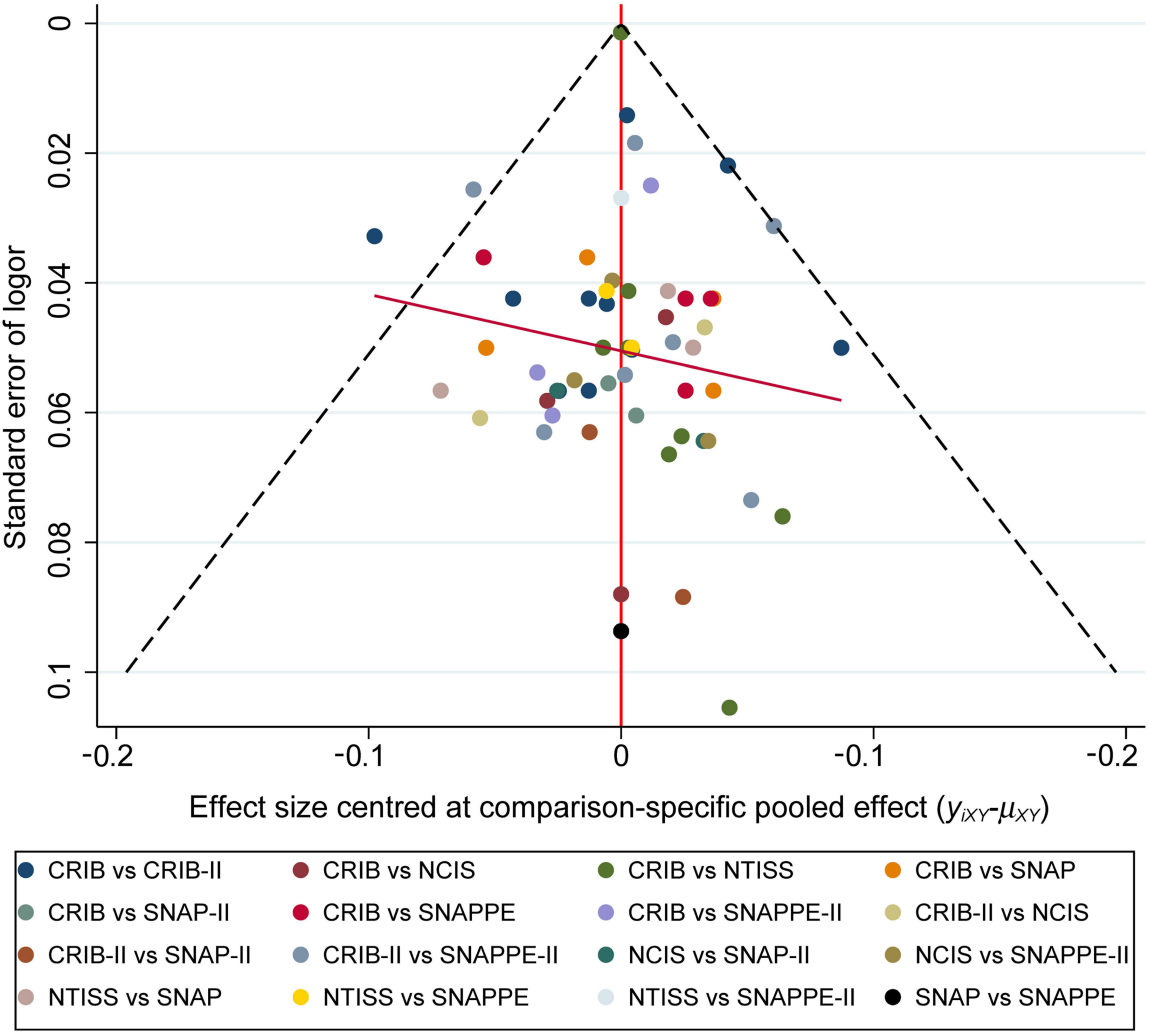
Funnel plot of the network meta-analysis.

The included literature was assessed for publication bias, and a corrected funnel plot was drawn, which is shown in Figure 4. The studies were evenly distributed on both sides of the center line of the funnel plot, which was symmetrical, and no significant publication bias was found.

## 4. Discussion

The present study compared the predictive values of critical illness scoring systems for preterm infant mortality risk using a network meta-analysis. A total of 19 articles involving 8 scoring systems were included in this study. According to the SUCRA ranking results, CRIB had the best predictive performance (SUCRA = 0.980). This study suggests that CRIB may be the most suitable scoring system for predicting the risk of preterm infant death.

The CRIB was formulated by Cockburn et al. (26) in 1993 and is suitable for preterm infants with a gestational age <31 weeks or a birth weight ≤1,500 g. The system includes six scoring items: gestational age, birth weight, congenital malformations, maximum base excess, and maximum and minimum fractions of inspired oxygen. Within 12 h after birth, the medical staff spend 5–10 min completing the assessment. Studies have shown that gestational age and birth weight are independent risk factors for preterm infant mortality, and with younger gestational age and lower birth weight comes a higher mortality rate (27). Additionally, the fraction of inspired oxygen (FiO_2_) reflects the maturity of lung development in preterm infants at birth. The imperfect development of lung function can lead to a lack of pulmonary surfactant and a lag in alveolar and pulmonary vascular development; therefore, it is necessary to increase the oxygen concentration FiO_2_ and provide alternative therapy to improve the blood gas index and oxygenation state. At the same time, the imperfect development of lung function makes infants vulnerable to invasion by many pathogenic factors, such as respiratory distress syndrome, bronchopulmonary dysplasia, infectious pneumonia, respiratory failure, and other complications (15, 28). Maximum base excess is an index used to assess the metabolic acid-base imbalance in the body, which can effectively reflect the steady state of the internal environment and is a necessary condition for normal life activities of the body. The primary increase or decrease of H + and HCO3- in preterm infants can cause acidosis or alkalosis. In severe cases, organ dysfunction, acute renal insufficiency, and shock, which are serious threats to life, may occur (7, 29). It can be observed that the six-item index of CRIB is an important evaluation index of clinical outcomes of preterm infants and has a strong predictive ability for the risk of death in preterm infants. Saravi et al. (30) compared the three scoring systems, CRIB, SNAP, and SNAPPE, in a retrospective analysis and found that CRIB had the highest predictive value for the mortality risk before discharge in preterm infants with birth weight ≤1,500 g, which was similar to the results of the present study. Nonetheless, CRIB has some limitations. For example, the maximum and minimum fractions of inspired oxygen in the evaluation index are easily influenced by ventilation mode and preventive application of pulmonary surfactant, and oxygen inhalation concentration values are determined by clinicians, leading to a lack of objectivity (15). Parry et al. (31) simplified and revised CRIB to obtain CRIB-II in 2003; the scoring indicators include birth weight, gestational age, temperature, sex, and maximum base excess, which are also suitable for preterm infants with a gestational age <31 weeks or a birth weight ≤1,500 g. However, compared with CRIB, the predictive efficacy of CRIB-II for the mortality risk in preterm infants is reduced (SUCRA = 0.525) (4). Although gestational age and birth weight are independent predictors of preterm infant mortality risk, CRIB's assessment of congenital malformations and lung maturity can further enhance its ability to predict mortality risk in preterm infants.

SNAP, formulated by Richardson et al. (32) in 1993, includes 28 scoring indexes, such as heart rate within 24 h after birth, blood pressure, oxygenation index, arterial partial pressure of oxygen (PaO_2_), creatinine, and blood urea nitrogen, and is suitable for newborns of various birth weights. In the same year, Richardson et al. (33) studied 1,621 newborns at three neonatal intensive care units in the United States. The results confirmed that birth weight, 5-min Apgar score, and small for gestational age were independent predictors of neonatal mortality risk. These three perinatal indicators were added to SNAP and developed into SNAPPE, which made the scoring system more comprehensive and effective. In 2001, Richardson et al. (34) simplified and corrected the scoring indicators based on SNAP and SNAPPE to obtain SNAP-II and SNAPPE-II. The SNAP-II scoring indicators were simplified to six indicators closely related to the risk of neonatal death. The lowest temperature, serum pH, PaO_2_/FiO_2_ ratio, multiple seizures, urine output, and lowest blood pressure were observed in the first 12 h after admission. SNAPPE-II was formed by including three perinatal indicators based on SNAP-II. The results of this present study showed that SNAPPE had a good predictive effect on mortality risk in preterm infants (SUCRA = 0.718). The possible reasons for this are as follows: the SNAPPE score is comprehensive, and the indicators involve various organ systems, including more clinical and laboratory parameters, which can better reflect the clinical status of preterm infants. Gestational age and birth weight can be used to evaluate the growth and development of preterm infants. The 5-min Apgar score can be used to preliminarily assess asphyxia and disease severity in preterm infants. The respiratory frequency and oxygenation index can reflect pulmonary oxygenation and ventilation function in preterm infants. Blood urea nitrogen and creatinine levels can be used to diagnose renal function diseases, bilirubin is an essential index for evaluating abnormal liver function, and blood sodium and potassium are mainly used to maintain intracellular and extracellular osmotic pressure and acid-base balance, reflecting the homeostasis of the internal environment. Therefore, a perfect scoring index makes the SNAPPE evaluation more accurate; however, the disadvantage is that data collection is cumbersome, and the acquisition of some items depends on laboratory inspection, which increases the difficulty of evaluation. The results of this present study also showed that compared with SNAP and SNAP-II, SNAPPE and SNAPPE-II could predict the mortality risk in preterm infants more accurately after adding the three perinatal indicators of birth weight, 5-min Apgar score, and small for gestational age. The results further confirmed that it is feasible to improve the accuracy of prediction by adding reliable scoring items, but it is also necessary to fully consider realistic conditions and operability and prioritize the selection of more sensitive and simple indicators to optimize the scoring system (11, 35).

The NCIS was formulated by the Chinese Medical Association in 2001 and is used to evaluate the condition of critically ill newborns and predict the risk of death. A total of 11 scoring indicators were used, as follows: heart rate, blood pressure, respiratory frequency, PaO_2_, pH, sodium, potassium, creatinine, blood urea nitrogen, hematocrit, and gastrointestinal condition (36). The results of the present study showed that the ability of NCIS to predict the mortality risk of preterm infants was limited (SUCRA = 0.422), and it could not accurately assess the mortality risk in preterm infants, which is consistent with the findings of previous studies (30, 37, 38). Such findings may be related to the fact that the NCIS score is mainly formulated with reference to full-term infants, and its evaluation index is not targeted at predicting the condition of preterm infants (36). Additionally, some NCIS scoring indicators may not be sufficiently accurate. For example, PaO_2_/FiO_2_ can more accurately reflect the respiratory function status of infants than PaO_2_, while cystatin-C and neutrophil gelatinase-associated lipocalin can more accurately reflect dynamic changes in renal function in infants than blood urea nitrogen and creatinine (10). Yang et al. (7) retrospectively analyzed 192 cases of extremely low birth weight infants and found that compared with CRIB, CRIB-II, SNAP-II, and SNAPPE-II, NCIS had the weakest correlation with birth weight and gestational age, which may be one of the reasons for its low predictive value. The NCIS scoring index must be further optimized to meet clinical needs.

NTISS, formulated by Gray et al. (39) in 1992, assesses the risk of neonatal illness and death based on treatment intensity, and includes 62 indicators, such as cardiopulmonary resuscitation, mechanical ventilation, extracorporeal membrane oxygenation, minor operation, major operation, dialysis, red blood cell transfusion, antibiotics, and insulin levels. The results of the current study revealed that the NTISS's ability to predict the mortality risk in preterm infants was weak (SUCRA = 0.478) and it was unable to objectively reflect the actual situation, which is consistent with the results of Oygur et al. (40). NTISS score indicators are based on treatment intensity to assess the mortality risk of preterm infants; however, therapeutic intervention measures may be affected by individual differences, hospitalization time, illness changes, medical equipment, and technical level and cannot fully reflect the real situation of infants (5).

Collectively, the present findings indicate that compared with other scoring systems, CRIB was more sensitive in predicting mortality risk in preterm infants, and its scoring items were sensitive indicators reflecting the condition of preterm infants. The other scoring systems have some shortcomings. For example, the SNAP, SNAPPE, and NTISS have a certain predictive value for mortality risk in preterm infants, but they have many scoring items and strict quality control of indicators. However, the equipment and instruments in grassroot hospitals are relatively simple, laboratory detection indicators are imperfect, and neonatal intensive care unit medical staff perform complex rescue tasks and often cannot evaluate them immediately. Hence, there are significant limitations to the popularization of clinical applications. Although CRIB-II, SNAP-II, and SNAPPE-II are simplified based on the original scoring system, which makes the scoring data easier to obtain and the evaluation process simpler, the predictive efficacy of the mortality risk in preterm infants is also reduced. NCIS is mainly formulated for term infants, and some indicators are not sufficiently accurate; therefore, it needs further optimization.

### 4.1. Study limitations

In this study, the network meta-analysis results were reliable, but several potential limitations should be noted. First, only published Chinese and English studies were included in this study, and other studies in other languages may have been missed. Second, some scoring systems in our network meta-analysis included only a few studies and studies with greater population coverage are needed in the future. Third, the included studies from different countries may have increased clinical heterogeneity. Finally, there were differences in the causes of death and evaluation times of preterm infants in the included studies, which may have affected the results.

## 5. Conclusions

The present results showed that CRIB had the best predictive efficiency for mortality risk in preterm infants and was simple to perform and easy to collect data. Thus, CRIB is recommended as the first choice for predicting the mortality risk in preterm neonates; however, the results still need to be verified using a larger number of samples. Multicenter studies should be conducted in the future to further compare the accuracy and application of various scoring systems for predicting the mortality risk in preterm infants.

## Data Availability

The datasets presented in this study can be found in online repositories. The names of the repository/repositories and accession number(s) can be found in the article/**Supplementary Material**.
