# Supplementary material for: Comparing different scoring systems for predicting mortality risk in preterm infants: a systematic review and network meta-analysis
Source: Front Pediatr. 2023 Dec 15;11:1287774. doi: 10.3389/fped.2023.1287774 (PMC10757321; doi:10.3389/fped.2023.1287774)
Supplement: Supplementary file 1 [file Datasheet1.docx]

Network meta-analysis with Stata software, the analysis results are as follows:


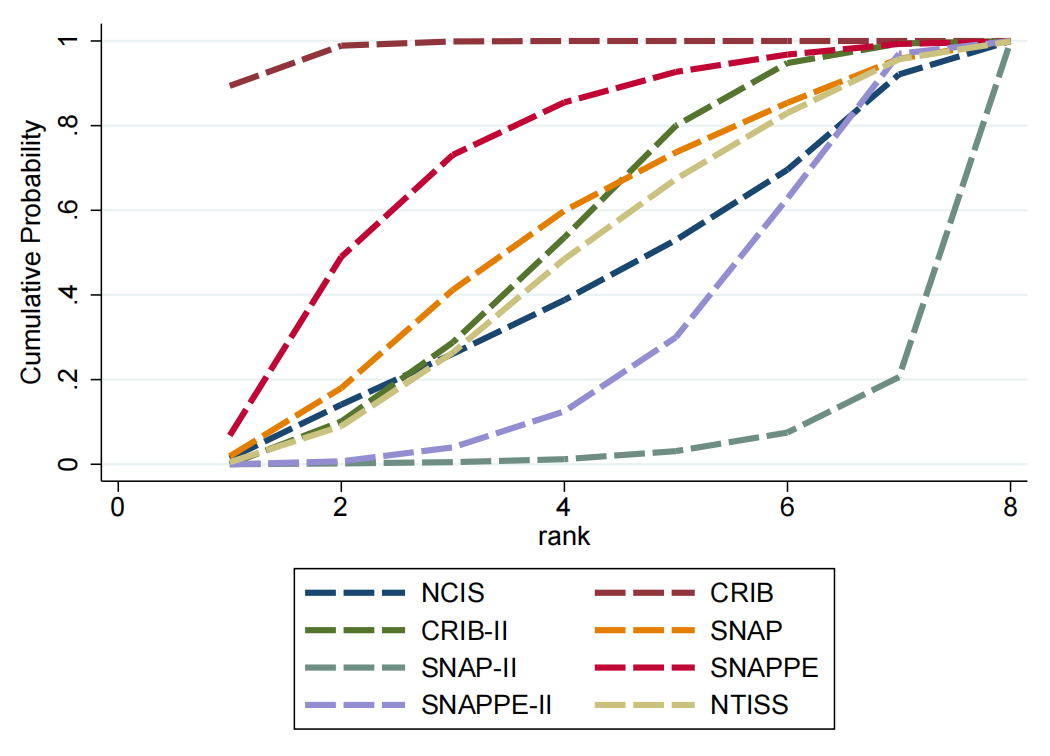


**Supplement Figure 1** SUCRA curve of predictive value of the eight scoring systems.

The SUCRA values of the eight scoring systems for predicting mortality risk in preterm infants were as follows: CRIB (0.983) > SNAPPE (0.719) > SNAP (0.537) > CRIB-Ⅱ (0.524) > NTISS (0.472) > NCIS (0.422) > SNAPPE-Ⅱ (0.296) > SNAP-Ⅱ (0.048).

**The analysis results of R software and Stata software are consistent**

**Supplement Table 1** Results of indirect comparisons of the scoring systems.

| **CRIB** | - | - | - | - | - | - | - |
| --- | --- | --- | --- | --- | --- | --- | --- |
| 1.06  (1.02,1.09) | **CRIB-Ⅱ** | - | - | - | - | - | - |
| 1.06  (1.01,1.13) | 1.01  (0.95,1.07) | **NCIS** | **-** | **-** | **-** | **-** | **-** |
| 1.06  (1.01,1.11) | 1.00  (0.95,1.05) | 0.99  (0.93,1.06) | **NTISS** | **-** | **-** | **-** | **-** |
| 1.05  (1.00,1.10) | 1.00  (0.94,1.05) | 0.99  (0.92,1.06) | 0.99  (0.94,1.05) | **SNAP** | **-** | **-** | **-** |
| 1.11  (1.05,1.17) | 1.05  (1.00,1.10) | 1.04  (0.98,1.11) | 1.05  (0.99,1.12) | 1.05  (0.99,1.13) | **SNAP-Ⅱ** | **-** | **-** |
| 1.04  (0.99,1.08) | 0.98  (0.93,1.03) | 0.97  (0.91,1.04) | 0.98  (0.93,1.03) | 0.98  (0.93,1.04) | 0.93  (0.87,1.00) | **SNAPPE** | **-** |
| 1.07  (1.04,1.11) | 1.02  (0.99,1.05) | 1.01  (0.95,1.06) | 1.02  (0.97,1.07) | 1.02  (0.96,1.08) | 0.97  (0.93,1.01) | 1.04  (0.98,1.09) | **SNAPPE-Ⅱ** |

The network meta-analysis showed that compared with CRIB-II, NCIS, NTISS, SNAP, SNAP-Ⅱ, and SNAPPE-II, CRIB was more effective in predicting the mortality risk in preterm infants (P<0.05). Relative to CRIB, and CRIB-II, SNAP-Ⅱ was worse in predicting the mortality risk in preterm infants (P<0.05). There were no statistically significant differences between the other scoring systems (P>0.05).

**The analysis results of R software and Stata software are consistent**
