# Supplementary material for: Comparing different scoring systems for predicting mortality risk in preterm infants: a systematic review and network meta-analysis
Source: Front Pediatr. 2023 Dec 15;11:1287774. doi: 10.3389/fped.2023.1287774 (PMC10757321; doi:10.3389/fped.2023.1287774)
Supplement: Supplementary file 2 [file Datasheet2.docx]

**Supplementary Tables**

**Table S1.** Details of the search strategy **(Web of Science)**

| **Search Details** |
| --- |
| #1 (((((TS=(preterm infant)) OR TS=(premature)) OR TS=(low birth weight)) OR TS=(LBW)) OR TS=(very low birth weight)) OR TS=(VLBW) and Preprint Citation Index (Exclude – Database) |
| #2 ((((((((((((((TS=(neonatal critical illness score)) OR TS=(NCIS)) OR TS=(neonatal therapeutical intervention score system)) OR TS=(NTISS)) OR TS=(clinical risk index for babies)) OR TS=(CRIB)) OR TS=(clinical risk index for babies II)) OR TS=(CRIB-II)) OR TS=(score for neonatal acute physiology perinatal extension)) OR TS=(score for neonatal acute physiology perinatal extension II)) OR TS=(SNAPPE-II)) OR TS=(score for neonatal acute physiology)) OR TS=(SNAP)) OR TS=(score for neonatal acute physiology II)) OR TS=(SNAP-II) and Preprint Citation Index (Exclude – Database) |
| #3 #1 AND #2 and Preprint Citation Index (Exclude – Database) |

| Entitlements | # | Search Query | Database | Results | Date Run |
| --- | --- | --- | --- | --- | --- |
| - WOS: 1900 to 2023 - DIIDW: 1966 to 2023 - INSPEC: 1969 to 2023 - KJD: 1980 to 2023 - MEDLINE: 1950 to 2023 - PPRN: 1991 to 2023 - PQDT: 1637 to 2023 - SCIELO: 2002 to 2023 | 1 | (((((TS=(preterm infant)) OR TS=(premature)) OR TS=(low birth weight)) OR TS=(LBW)) OR TS=(very low birth weight)) OR TS=(VLBW) and Preprint Citation Index (Exclude – Database) | All Databases | 472019 | Tue Nov 28 2023 22:54:34 GMT+0800 (中国标准时间) |
| - WOS: 1900 to 2023 - DIIDW: 1966 to 2023 - INSPEC: 1969 to 2023 - KJD: 1980 to 2023 - MEDLINE: 1950 to 2023 - PPRN: 1991 to 2023 - PQDT: 1637 to 2023 - SCIELO: 2002 to 2023 | 2 | ((((((((((((((TS=(neonatal critical illness score)) OR TS=(NCIS)) OR TS=(neonatal therapeutical intervention score system)) OR TS=(NTISS)) OR TS=(clinical risk index for babies)) OR TS=(CRIB)) OR TS=(clinical risk index for babies II)) OR TS=(CRIB-II)) OR TS=(score for neonatal acute physiology perinatal extension)) OR TS=(score for neonatal acute physiology perinatal extension II)) OR TS=(SNAPPE-II)) OR TS=(score for neonatal acute physiology)) OR TS=(SNAP)) OR TS=(score for neonatal acute physiology II)) OR TS=(SNAP-II) and Preprint Citation Index (Exclude – Database) | All Databases | 200235 | Tue Nov 28 2023 22:56:44 GMT+0800 (中国标准时间) |
| - WOS: 1900 to 2023 - DIIDW: 1966 to 2023 - INSPEC: 1969 to 2023 - KJD: 1980 to 2023 - MEDLINE: 1950 to 2023 - PPRN: 1991 to 2023 - PQDT: 1637 to 2023 - SCIELO: 2002 to 2023 | 3 | #1 AND #2 and Preprint Citation Index (Exclude – Database) | All Databases | 1802 | Tue Nov 28 2023 22:56:58 GMT+0800 (中国标准时间) |

**Table S2.** Details of the search strategy **(PubMed)**

| **Search Details** |
| --- |
| #1 ((((("preterm infant"[MeSH Terms]) OR (premature)) OR (low birth weight)) OR (LBW)) OR (very low birth weight)) OR (VLBW) |
| #2 ((((((((((((((("neonatal critical illness score"[MeSH Terms]) OR (NCIS)) OR ("neonatal therapeutical intervention score system"[MeSH Terms])) OR (NTISS)) OR ("clinical risk index for babies"[MeSH Terms])) OR (CRIB)) OR ("clinical risk index for babies II"[MeSH Terms])) OR (CRIB-II)) OR ("score for neonatal acute physiology perinatal extension"[MeSH Terms])) OR (SNAPPE)) OR ("score for neonatal acute physiology perinatal extension II"[MeSH Terms])) OR (SNAPPE-II)) OR ("score for neonatal acute physiology"[MeSH Terms])) OR (SNAP)) OR ("score for neonatal acute physiology II"[MeSH Terms])) OR (SNAP-II) |
| #3 #1 AND #2 |

| Search  number | Query | Sort By | Filters | Search Details | Results | Time |
| --- | --- | --- | --- | --- | --- | --- |
| 1 | ((((("preterm infant"[MeSH Terms]) OR (premature)) OR (low birth weight)) OR (LBW)) OR (very low birth weight)) OR (VLBW) | Publication Date |  | "premature birth"[MeSH Terms] OR ("premature"[All Fields] AND "birth"[All Fields]) OR "premature birth"[All Fields] OR "premature"[All Fields] OR "prematurely"[All Fields] OR "prematures"[All Fields] OR "prematurities"[All Fields] OR "prematurity"[All Fields] OR ("infant, low birth weight"[MeSH Terms] OR ("infant"[All Fields] AND "low"[All Fields] AND "birth"[All Fields] AND "weight"[All Fields]) OR "low birth weight infant"[All Fields] OR ("low"[All Fields] AND "birth"[All Fields] AND "weight"[All Fields]) OR "low birth weight"[All Fields]) OR "LBW"[All Fields] OR ("infant, very low birth weight"[MeSH Terms] OR ("infant"[All Fields] AND "low"[All Fields] AND "birth"[All Fields] AND "weight"[All Fields]) OR "very low birth weight infant"[All Fields] OR ("low"[All Fields] AND "birth"[All Fields] AND "weight"[All Fields]) OR "very low birth weight"[All Fields]) OR "VLBW"[All Fields] | 291,182 | 9:45:53 |
| 2 | ((((((((((((((("neonatal critical illness score"[MeSH Terms]) OR (NCIS)) OR ("neonatal therapeutical intervention score system"[MeSH Terms])) OR (NTISS)) OR ("clinical risk index for babies"[MeSH Terms])) OR (CRIB)) OR ("clinical risk index for babies II"[MeSH Terms])) OR (CRIB-II)) OR ("score for neonatal acute physiology perinatal extension"[MeSH Terms])) OR (SNAPPE)) OR ("score for neonatal acute physiology perinatal extension II"[MeSH Terms])) OR (SNAPPE-II)) OR ("score for neonatal acute physiology"[MeSH Terms])) OR (SNAP)) OR ("score for neonatal acute physiology II"[MeSH Terms])) OR (SNAP-II) | Publication Date |  | "NCIS"[All Fields] OR "NTISS"[All Fields] OR ("infant equipment"[MeSH Terms] OR ("infant"[All Fields] AND "equipment"[All Fields]) OR "infant equipment"[All Fields] OR "crib"[All Fields]) OR "CRIB-II"[All Fields] OR "SNAPPE"[All Fields] OR "SNAPPE-II"[All Fields] OR "SNAP"[All Fields] OR "SNAP-II"[All Fields] | 29,010 | 9:46:08 |
| 3 | (((((("preterm infant"[MeSH Terms]) OR (premature)) OR (low birth weight)) OR (LBW)) OR (very low birth weight)) OR (VLBW)) AND (((((((((((((((("neonatal critical illness score"[MeSH Terms]) OR (NCIS)) OR ("neonatal therapeutical intervention score system"[MeSH Terms])) OR (NTISS)) OR ("clinical risk index for babies"[MeSH Terms])) OR (CRIB)) OR ("clinical risk index for babies II"[MeSH Terms])) OR (CRIB-II)) OR ("score for neonatal acute physiology perinatal extension"[MeSH Terms])) OR (SNAPPE)) OR ("score for neonatal acute physiology perinatal extension II"[MeSH Terms])) OR (SNAPPE-II)) OR ("score for neonatal acute physiology"[MeSH Terms])) OR (SNAP)) OR ("score for neonatal acute physiology II"[MeSH Terms])) OR (SNAP-II)) | Publication Date |  | ("premature birth"[MeSH Terms] OR ("premature"[All Fields] AND "birth"[All Fields]) OR "premature birth"[All Fields] OR "premature"[All Fields] OR "prematurely"[All Fields] OR "prematures"[All Fields] OR "prematurities"[All Fields] OR "prematurity"[All Fields] OR ("infant, low birth weight"[MeSH Terms] OR ("infant"[All Fields] AND "low"[All Fields] AND "birth"[All Fields] AND "weight"[All Fields]) OR "low birth weight infant"[All Fields] OR ("low"[All Fields] AND "birth"[All Fields] AND "weight"[All Fields]) OR "low birth weight"[All Fields]) OR "LBW"[All Fields] OR ("infant, very low birth weight"[MeSH Terms] OR ("infant"[All Fields] AND "low"[All Fields] AND "birth"[All Fields] AND "weight"[All Fields]) OR "very low birth weight infant"[All Fields] OR ("low"[All Fields] AND "birth"[All Fields] AND "weight"[All Fields]) OR "very low birth weight"[All Fields]) OR "VLBW"[All Fields]) AND ("NCIS"[All Fields] OR "NTISS"[All Fields] OR ("infant equipment"[MeSH Terms] OR ("infant"[All Fields] AND "equipment"[All Fields]) OR "infant equipment"[All Fields] OR "crib"[All Fields]) OR "CRIB-II"[All Fields] OR "SNAPPE"[All Fields] OR "SNAPPE-II"[All Fields] OR "SNAP"[All Fields] OR "SNAP-II"[All Fields]) | 1,970 | 9:58:16 |
| 4 | (((((("preterm infant"[MeSH Terms]) OR (premature)) OR (low birth weight)) OR (LBW)) OR (very low birth weight)) OR (VLBW)) AND (((((((((((((((("neonatal critical illness score"[MeSH Terms]) OR (NCIS)) OR ("neonatal therapeutical intervention score system"[MeSH Terms])) OR (NTISS)) OR ("clinical risk index for babies"[MeSH Terms])) OR (CRIB)) OR ("clinical risk index for babies II"[MeSH Terms])) OR (CRIB-II)) OR ("score for neonatal acute physiology perinatal extension"[MeSH Terms])) OR (SNAPPE)) OR ("score for neonatal acute physiology perinatal extension II"[MeSH Terms])) OR (SNAPPE-II)) OR ("score for neonatal acute physiology"[MeSH Terms])) OR (SNAP)) OR ("score for neonatal acute physiology II"[MeSH Terms])) OR (SNAP-II)) | Publication Date | Full text | (("premature birth"[MeSH Terms] OR ("premature"[All Fields] AND "birth"[All Fields]) OR "premature birth"[All Fields] OR "premature"[All Fields] OR "prematurely"[All Fields] OR "prematures"[All Fields] OR "prematurities"[All Fields] OR "prematurity"[All Fields] OR ("infant, low birth weight"[MeSH Terms] OR ("infant"[All Fields] AND "low"[All Fields] AND "birth"[All Fields] AND "weight"[All Fields]) OR "low birth weight infant"[All Fields] OR ("low"[All Fields] AND "birth"[All Fields] AND "weight"[All Fields]) OR "low birth weight"[All Fields]) OR "LBW"[All Fields] OR ("infant, very low birth weight"[MeSH Terms] OR ("infant"[All Fields] AND "low"[All Fields] AND "birth"[All Fields] AND "weight"[All Fields]) OR "very low birth weight infant"[All Fields] OR ("low"[All Fields] AND "birth"[All Fields] AND "weight"[All Fields]) OR "very low birth weight"[All Fields]) OR "VLBW"[All Fields]) AND ("NCIS"[All Fields] OR "NTISS"[All Fields] OR ("infant equipment"[MeSH Terms] OR ("infant"[All Fields] AND "equipment"[All Fields]) OR "infant equipment"[All Fields] OR "crib"[All Fields]) OR "CRIB-II"[All Fields] OR "SNAPPE"[All Fields] OR "SNAPPE-II"[All Fields] OR "SNAP"[All Fields] OR "SNAP-II"[All Fields])) AND (fft[Filter]) | 1,644 | 9:59:00 |
